# Copy number variation in the speciation of pigs: a possible prominent role for olfactory receptors

**DOI:** 10.1186/s12864-015-1449-9

**Published:** 2015-04-22

**Authors:** Yogesh Paudel, Ole Madsen, Hendrik-Jan Megens, Laurent A F Frantz, Mirte Bosse, Richard P M A Crooijmans, Martien A M Groenen

**Affiliations:** Animal Breeding and Genomics Centre, Wageningen University, 6700 AH Wageningen, The Netherlands; Current address: Roche Pharma Research and Early Development, Roche Innovation Center Basel, F. Hoffmann-La Roche Ltd, 4070 Basel, Switzerland

**Keywords:** Speciation, Structural variation, Copy number variation, Next generation sequencing data, Read depth method

## Abstract

**Background:**

Unraveling the genetic mechanisms associated with reduced gene flow between genetically differentiated populations is key to understand speciation. Different types of structural variations (SVs) have been found as a source of genetic diversity in a wide range of species. Previous studies provided detailed knowledge on the potential evolutionary role of SVs, especially copy number variations (CNVs), between well diverged species of e.g. primates. However, our understanding of their significance during ongoing speciation processes is limited due to the lack of CNV data from closely related species. The genus *Sus* (pig and its close relatives) which started to diverge ~4 Mya presents an excellent model for studying the role of CNVs during ongoing speciation.

**Results:**

In this study, we identified 1408 CNV regions (CNVRs) across the genus *Sus*. These CNVRs encompass 624 genes and were found to evolve ~2.5 times faster than single nucleotide polymorphisms (SNPs). The majority of these copy number variable genes are olfactory receptors (ORs) known to play a prominent role in food foraging and mate recognition in *Sus*. Phylogenetic analyses, including novel Bayesian analysis, based on CNVRs that overlap ORs retain the well-accepted topology of the genus *Sus* whereas CNVRs overlapping genes other than ORs show evidence for random drift and/or admixture.

**Conclusion:**

We hypothesize that inter-specific variation in copy number of ORs provided the means for rapid adaptation to different environments during the diversification of the genus *Sus* in the Pliocene. Furthermore, these regions might have acted as barriers preventing massive gene flow between these species during the multiple hybridization events that took place later in the Pleistocene suggesting a possible prominent role of ORs in the ongoing *Sus* speciation.

**Electronic supplementary material:**

The online version of this article (doi:10.1186/s12864-015-1449-9) contains supplementary material, which is available to authorized users.

## Background

Speciation is one of the major evolutionary drivers of the diversity of life on earth. Understanding the process by which populations diversify leading, ultimately, to speciation has been one of the major focuses of evolutionary biologists for decades [1-3]. Two major models of speciation have been put forward. The first model, also known as allopatric speciation, involves cessation of gene flow between two newly formed populations as a result of geographical isolation (i.e. mountain ranges, rivers). The second model, parapatric or sympatric speciation, involves cessation of gene flow between two populations with overlapping geographical range [4-6]. Many recent genetic studies, on organisms as diverse as fish [7], birds [8], insects [9,10], amphibians [6], mammals [11-13] and plants [14], have shown that genetic exchange during population diversification is more common than was originally anticipated. Hence, the reduction of gene flow between sub-populations or species, that inhabit the same geographic range, often involves a period of extrinsic reproductive isolation before acquiring an eventual intrinsic reproductive isolation.

The mechanisms by which gene flow reduces between diverging populations that overlap in their geographical range are still not very well understood. A major goal of geneticists and evolutionary biologists is to identify the mechanisms or genes and/or regions in the genome that are involved in the reduction of gene flow and eventual emergence of reproductive isolation between diverging populations. In animals, only a few genes have so far been identified to be involved in speciation, for example *Prdm9* in mouse [15], and *Odysseus*-*site homeobox* [16], *JYalpha* [17] and *GA19777 Overdrive* [18] in flies. These sparse examples of identified speciation genes do not seem to suggest a common or general universal pathway/process leading to speciation but rather point to the involvement of a variety of different mechanisms in the evolution of pre- and post-zygotic barriers between different species.

Speciation with gene flow could be achieved through the reduction of gene flow at specific loci in the genome, also coined islands of speciation [19,20]. Multiple studies have successfully identified possible islands of speciation in the genomes of diverging species [8,19]. However, the exact contribution of these regions in speciation is still to be unraveled. Furthermore, these studies have mainly focused on genetic variation due to single nucleotide polymorphisms (SNPs) and very few studies have investigated the role that structural variations (SVs) play in the process of population diversification [21,22]. Copy number variations (CNVs), a class of SVs, can be a major mechanism driving gene and genome evolution by duplicating and deleting segments of the genome and as a result, create novel gene functions, disrupt gene functions, or affect regulatory mechanisms in the genome. The majority of inter-species CNV studies have focused on primates [23-27] and suggested that species-specific copy numbers (CNs) can be evolutionarily favored because of their adaptive benefits [24,25,27-30]. However, these studies only provide insights into the role of CNVs between well-diverged species (i.e. Chimpanzees and Humans), making it difficult to determine whether these variations between species have arisen during speciation or rather accumulated during post-speciation.

The species of the genus *Sus* provide a good model to study the effect of CNV regions (CNVRs) in the process of speciation. Genus *Sus* comprises of at least seven morphologically and genetically well-defined species [31], that inhabit the five biodiversity hotspots in Islands and Mainland South East Asia (ISEA and MSEA) [32]. Recent findings showed that these species diverged during the late Pliocene (4–2.5 Mya), due to their isolation on different islands of ISEA and underwent multiple rounds of small scale inter-specific hybridization during the glacial periods of the Pleistocene (2.5-0.01 Mya) [31]. Indeed, the frequent occurrence of glacial periods during the Pleistocene, resulted in land bridges between ISEA and MSEA allowing migration between islands [31]. Therefore, the process of divergence between the pigs in ISEA and MSEA, effectively follows alternating periods of allopatric (warm periods) and parapatric (glacial periods) conditions. However, while these species can be identified based on morphology and/or DNA and are still capable of producing fertile offspring [33], the mechanisms that prevented these species from large scale homogenizing during the numerous glacial periods of the Pleistocene remain unclear.

In this study, we analyzed the complete genome sequence of four different species of the genus *Sus*, that are restricted to ISEA (*Sus*-ISEA): *Sus barbatus* (Bearded pig on Borneo), *Sus celebensis* (Sulawesi warty pig), *Sus cebifrons* (Philippine warty pig), *Sus verrucosus* (Javan warty pig) and three populations of the species *Sus scrofa* from Europe, China and Sumatra. We compared and contrasted the pattern of CNVs among population/species, in order to investigate the role that CNVRs may play in this on-going process of speciation.

## Results

**Figure 1:**
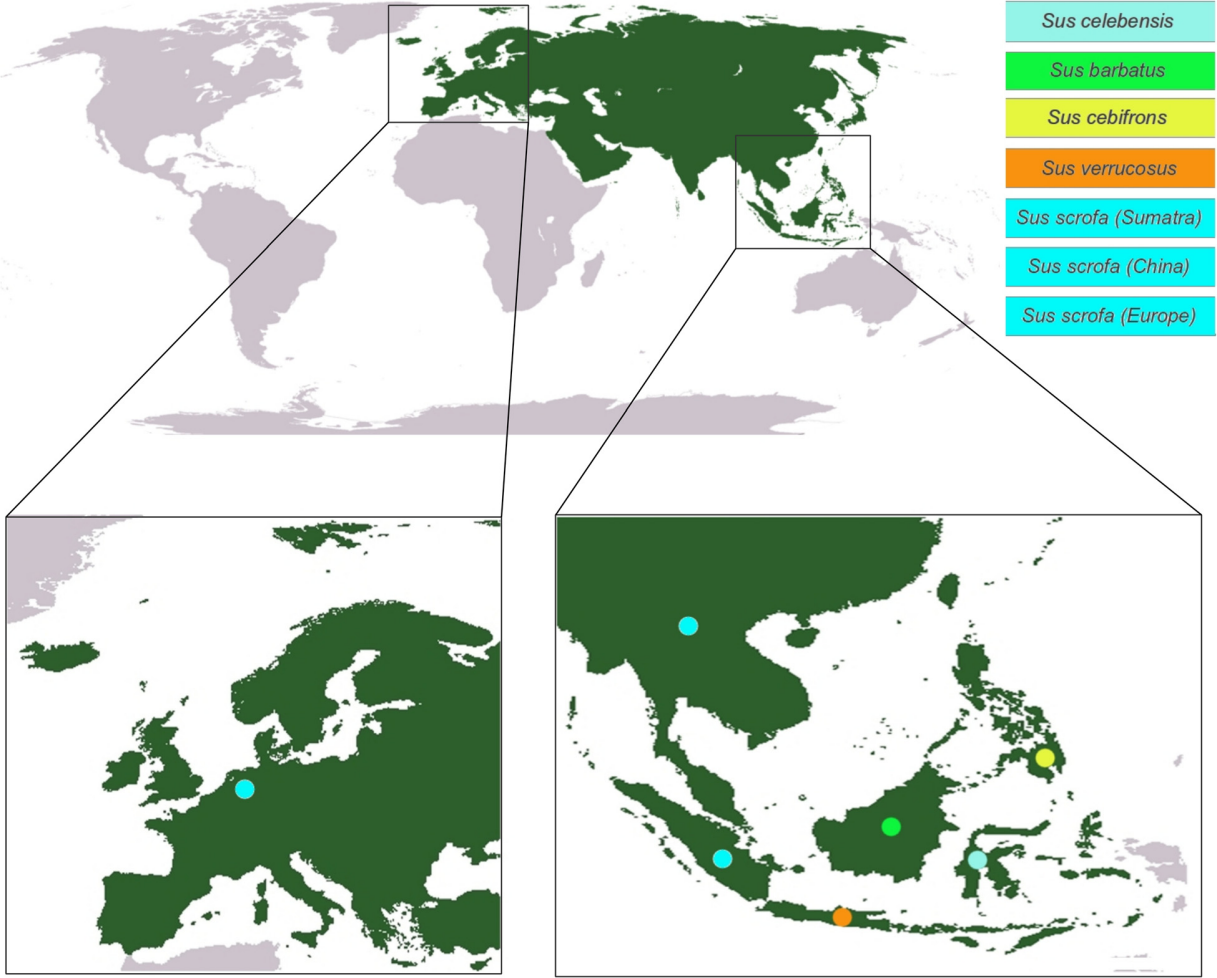
Schematic overview of origin of *Sus* populations across Eurasia and Island of South East Asia used in this study.

Whole genome re-sequencing data were obtained for seven populations (two individuals of the same species from ISEA; *Sus cebifrons* (critically endangered [34]), *Sus celebensis*, *Sus verrucosus* (endangered [34]) and *Sus barbatus* (in case of *Sus barbatus* we obtained data from four individuals) and two individuals each from three diverged populations of *Sus scrofa*; from Sumatra, China and Europe (Table 1, Figure 1, Additional file 1: Table S1A). Previous analyses have shown the read depth (RD) method to be an accurate method for computational detection of the CN of regions throughout the genome, especially with high coverage data [35-38]. Since our main goal was the identification of inter-population CNVRs, the two samples from the same population were combined. The combined data was used to identify inter-population CNVRs between the seven populations by aligning short reads to the *Sus scrofa* reference genome [39]. In the case of *Sus barbatus*, all possible pairwise combinations of the four individuals displayed a high level of congruence in CN detection in both intra- and inter-population comparison (data not shown). To avoid bias due to sampling size and total coverage we selected two of four *Sus barbatus* individuals in order to give a read coverage comparable with the other populations studied (Additional file 1: Table S1A). We tested the assumption that combining individuals from the same population would not create any significant bias due to the expected higher inter- than intra-population variation by comparing CN among and between the seven populations. We found that the copy number differences (CNDs) between pairs of individuals from different populations were significantly higher than between individuals from the same population (p-value <0.001, Wilcoxon test, Additional file 2: Figure S1A and S1B). Thus, combining two individuals of the same population, will likely result in a higher sensitivity in calling CN with a relative minimal bias in the inter-population comparison. For each population, multi copy regions (MCRs) were defined by applying a threshold of a minimum of 6 consecutive 1 kilobase (Kb) bins that have an average CN higher than 2.5. All the MCRs were then retrieved from all populations and we then chained MCRs that were (partially) overlapping between two or more populations. We computed the CN for all chained MCRs in each population and for each MCR, the standard deviation (s.d.) of CN between the seven populations was estimated. All MCRs with a s.d. ≥0.7 were regarded as CNVRs [38]. We identified 1408 regions, encompassing 17.83 megabases (Mb) on the *Sus scrofa* reference genome, as CNVRs (Additional file 1: Table S1B and S1C, Additional file 2: Figure S1) (see material and methods for details on detection of CN, MCR, and CNVR).

**Table 1:** **Read depth of individuals and grouped individuals used (information of other** ***Sus barbatus*** **individuals can be found in** Additional file 1: **Table S1A)**

**Names**	**Combined**	**Separate**	**Separate depth**	**Combined depth**
Sus barbatus	Sbar	Sbar1	9.087	17.186
		Sbar2	8.087	
Sus cebifrons	Sceb	Sceb1	9.36	18.6
		Sceb2	9.174	
Sus celebensis	Scel	Scel1	18.409	25.475
		Scel2	7.046	
Sus verrucosus	Sver	Sver1	9.088	18.844
		Sver2	10.127	
Sus scrofa	Sumatra	Sumatra1	10.961	22.247
		Sumatra2	11.113	
Sus scrofa	China	China1	7.965	19.172
		China2	11.268	
Sus scrofa	Europe	Europe1	7.555	18.529
		Europe2	11.056

**Figure 2:**
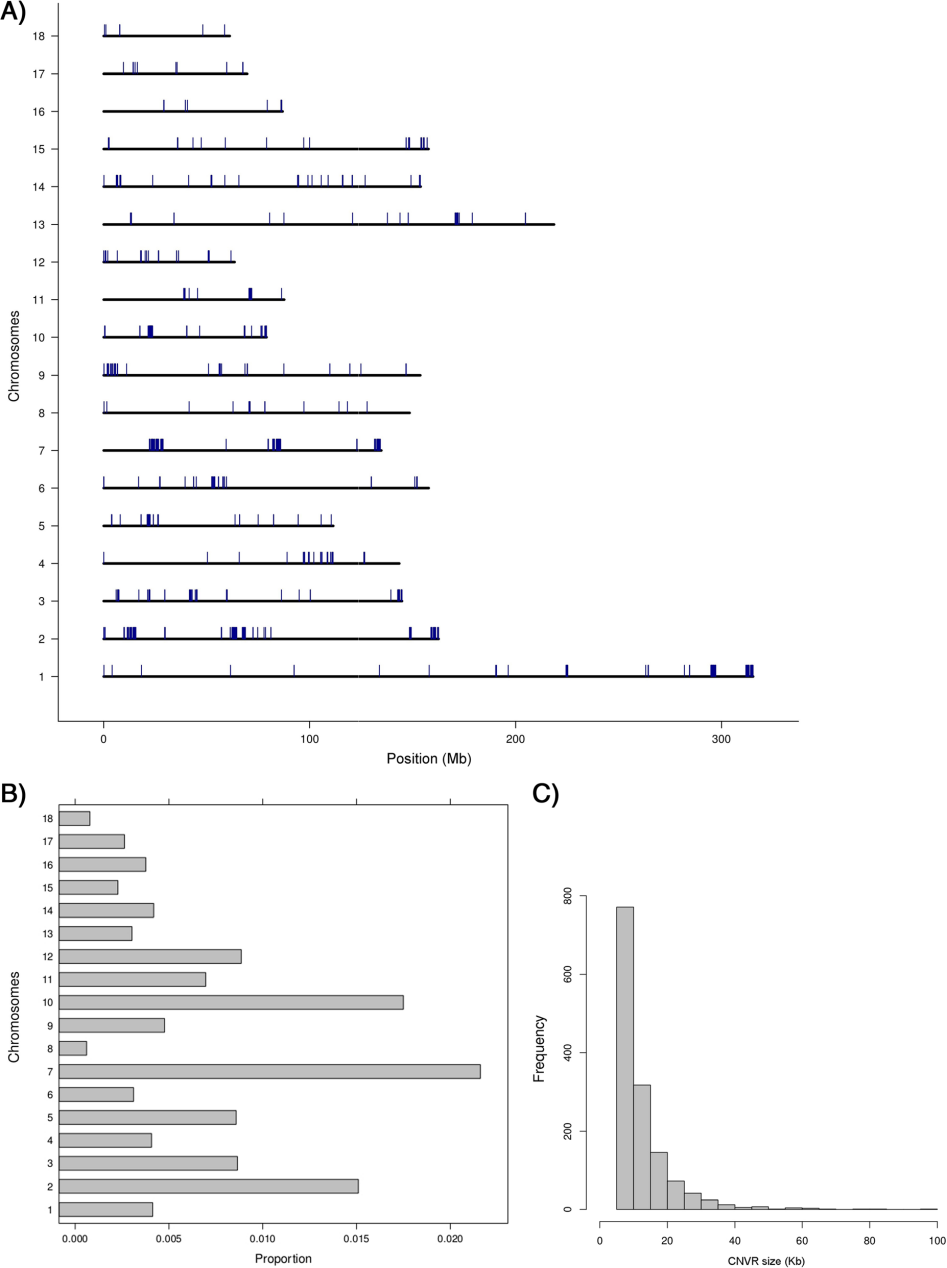
Distribution, proportion, and frequency of CNVRs in the pig genome. **A**: Distribution of CNVRs on the different chromosomes of the porcine genome. **B**: Proportion of CNVRs per chromosome. **C**: Frequency and size of CNVRs.

**Figure 3:**
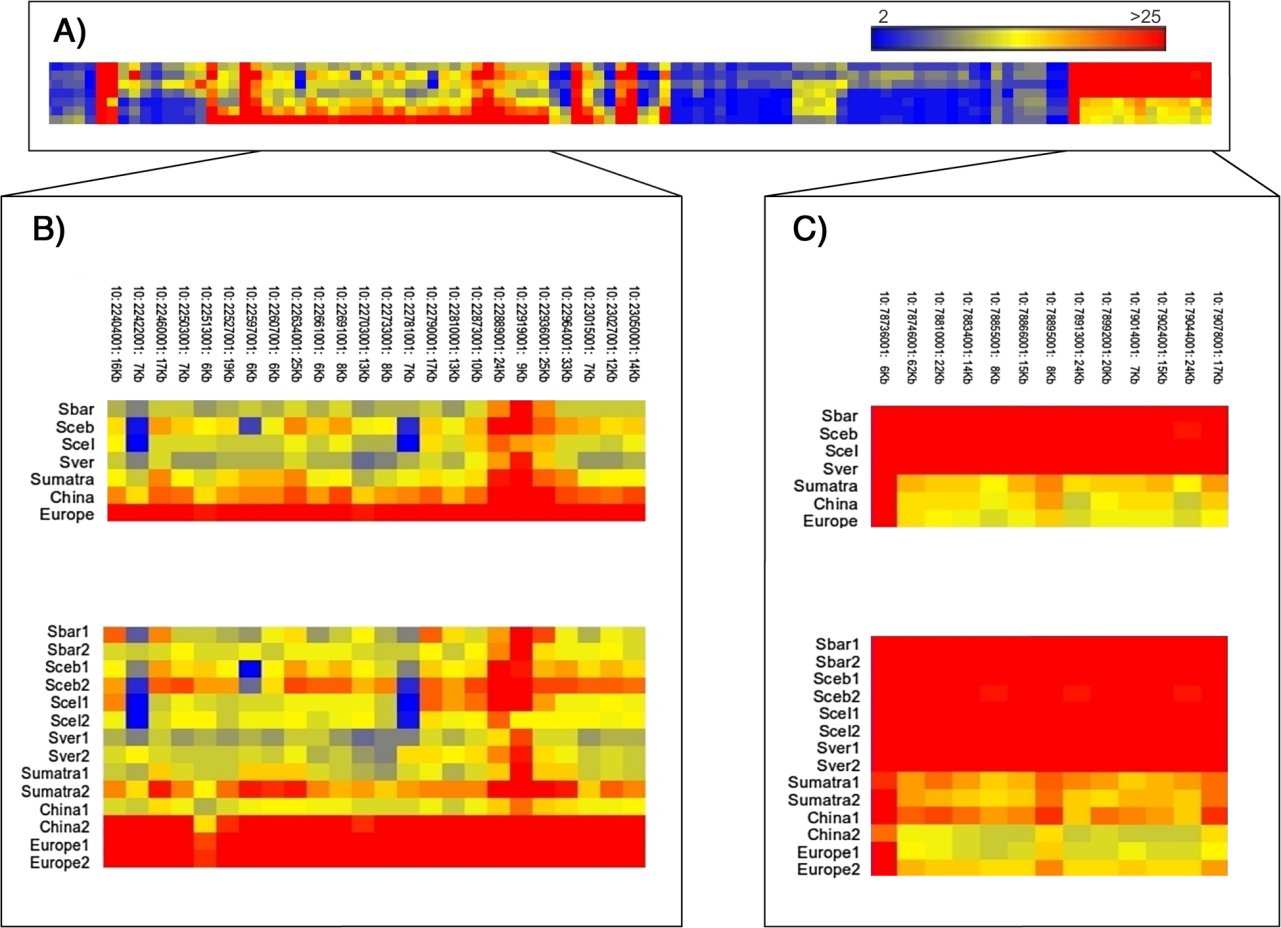
Heatmap of CNVRs. **A**: Heatmap of CNVRs on chromosome 10. Each column represents one CNVRs and each row represents a population. **B**: Heatmap of a 0.81 Mb region on chromosome 10 (SSC10: 22.24 Mb - 23.05 Mb; 24 CNVRs). Each column represents one CNVR (chromosome; CNVRs starting position; size of the CNVR) and each row represents one population (upper panel) or single individual (lower panel). Abbreviations: Sbar (*Sus barbatus*), Sceb (*Sus cebifrons*), Scel (*Sus celebensis*), Sver (*Sus verrucosus*), Sumatra (*Sus scrofa* population from Sumatra), China (*Sus scrofa* from China), Europe (*Sus scrofa* from Europe). **C**: Heatmap of a ~370 Kb region on chromosome 10 (SSC10: 78.7 Mb - 79.07 Mb; 13 CNVRs of different sizes. Each column represents one CNVRs (chromosome; CNVRs starting position; size of the CNVR) and each row represents one population (upper panel) or single individual (lower panel). Abbreviations: Sbar (*Sus barbatus*), Sceb (*Sus cebifrons*), Scel (*Sus celebensis*), Sver (*Sus verrucosus*), Sumatra (*Sus scrofa* population from Sumatra), China (*Sus scrofa* from China), Europe (*Sus scrofa* from Europe).

Although CNVRs were found on every chromosome, the number and the total size of CNVRs per chromosome are not correlated with chromosome length (Figure 2A and B), which is consistent with our previous study related to CNVRs in the porcine genome [38]. Many of the identified CNVRs are relatively small, close to the effective resolution of 6 Kb. While the size of CNVRs ranges from 6 to 98 Kb, the majority (1089 out of 1408; 78%) of the CNVRs that were identified is between 6 and 15 Kb (Figure 2C). We did not observe any CNVR larger than 98 Kb which is probably due to incompleteness and assembly errors in the current genome build resulting in gaps in the genome. In addition, the presence of repetitive elements may preclude the chaining of smaller segments of large CNVRs. Repetitive sequences will break the contiguity of defined CNVRs as those regions were masked in the genome prior to the alignment. We observed a number of regions on some chromosomes having cluster of CNVRs with comparatively higher CN in some populations. For example, the 0.81 Mb region between 22.24 Mb - 23.05 Mb on chromosome 10 (Figures 3A and B) shows higher CNs in the *Sus scrofa* populations (CN range in *Sus scrofa* 0 to 85; CN range in *Sus*-ISEA 0 to 39). Another example is the 370 Kb region between 78.7 Mb and 79.07 Mb on chromosome 10 (Figure 3A and C) that shows a series of regions with high CN in *Sus*-ISEA (CN range in *Sus*-ISEA 22 to 72; CN range in *Sus scrofa* 12 to 46).

Overall, most of the CNVRs identified displayed CN higher than two in all seven populations (1077 out of 1408 region) with only a small fraction (29; 211 Kb) being population specific. This could be due to the stringent criteria implemented to reduce false positive CNV calls. *Sus barbatus* showed the largest number of MCRs observed as variable in CN in all the seven populations (1358; 17.33 Mb) whereas *Sus scrofa* from Sumatra showed the lowest number of MCRs observed as variable in CN in all the seven populations (1197; 15.613 Mb) (Additional file 1: Table S1D).

### Experimental validations

We used quantitative real time-polymerase chain reaction (qPCR) to validate the identified CNVRs. We randomly selected ten genic CNVRs, ten non-genic CNVRs and five diploid regions and tested these using two distinct primer sets per locus. All 25 assays were successful and all 25 showed 100% agreement with our CNVRs predictions, indicating a low false discovery rate for calling CNVRs based on the RD analysis (Additional file 1: Table S1E).

### Functional relevance of CNVRs in the genus *Sus*

We used the porcine gene annotation of the current genome build (*Sus scrofa* build10.2, Ensembl release 75 [40]) to identify genes encompassing CNVRs. To improve the reliability of the functional annotation of CNVRs, only genes having at least 70 percent overlap with a CNVR were considered. The CN of the genes were set at the CN of the overlapping CNVRs. Out of the 21,630 protein coding genes annotated in the current genome build [39], 624 genes were found to overlap with 504 CNVRs (35.8% of total CNVRs) (Additional file 3: Table S2A).

The olfactory receptor gene family, one of the largest gene families in the porcine genome [29,39], is highly over-represented with 413 out of 624 genes overlapping a CNVR (Additional file 3: Table S2B). Genes involved in immune response, such as *IFN* (*Alpha*-*8*, *11*, *14*; *Delta*-*2*), *IFNW1*, *IGK* (*V1D*-*43*, *V2*-*28*), *IL1B* and *PG3I*, also show variation in CN between populations.

Only few genes exhibit a high CN in a single population or a general high number of copies with much variation in two or more population. For example, *PSMB5* shows higher CNs in *Sus*-ISEA (from 21 in *Sus celebensis* to 10 in *Sus cebifrons*) but no sign of duplication in the three population of *Sus scrofa* (1–2 copies). *NBPF6 and NBPF11* show high CN in all populations but with large variation in *Sus*-ISEA individuals (from 18 to 44 for *NBPF6* with s. d. of 11.1 and 21 to 60 for *NBPF11* with s. d. of 15.7). Likewise, *SAL1* shows CNV only between *Sus scrofa* populations (from 2–11 with s.d. of 3.48).

The porcine-specific immune-defense related genes *NPG3* and *PMAP23*, together with the other immune related genes *USP17L2*, *CDK20*, *POMC*, were found to be variable in CN with in general high variation in *Sus scrofa* populations. In addition, other previously identified CNV-genes in pigs involved in metabolism (*AMY1A*, *AMY2*, *AMY2A*, *AMY2B*) and detoxification (*UGT2B10*, *UGT1A3*, *CYPA11*, *CYPA22*, *CYP4F3* and *CYP4X1*) are found to be variable in CN in this study as well.

A gene ontology (GO) enrichment analysis on all 624 genes overlapping CNVRs revealed that most of these genes are involved in biological processes regulating sensory perception of smell (p < 0.001), signal transduction (p < 0.001), neurological process (p < 0.001) and metabolic process (p < 0.001) (Additional file 3: Table S2C).

### Cluster Analysis

**Figure 4:**
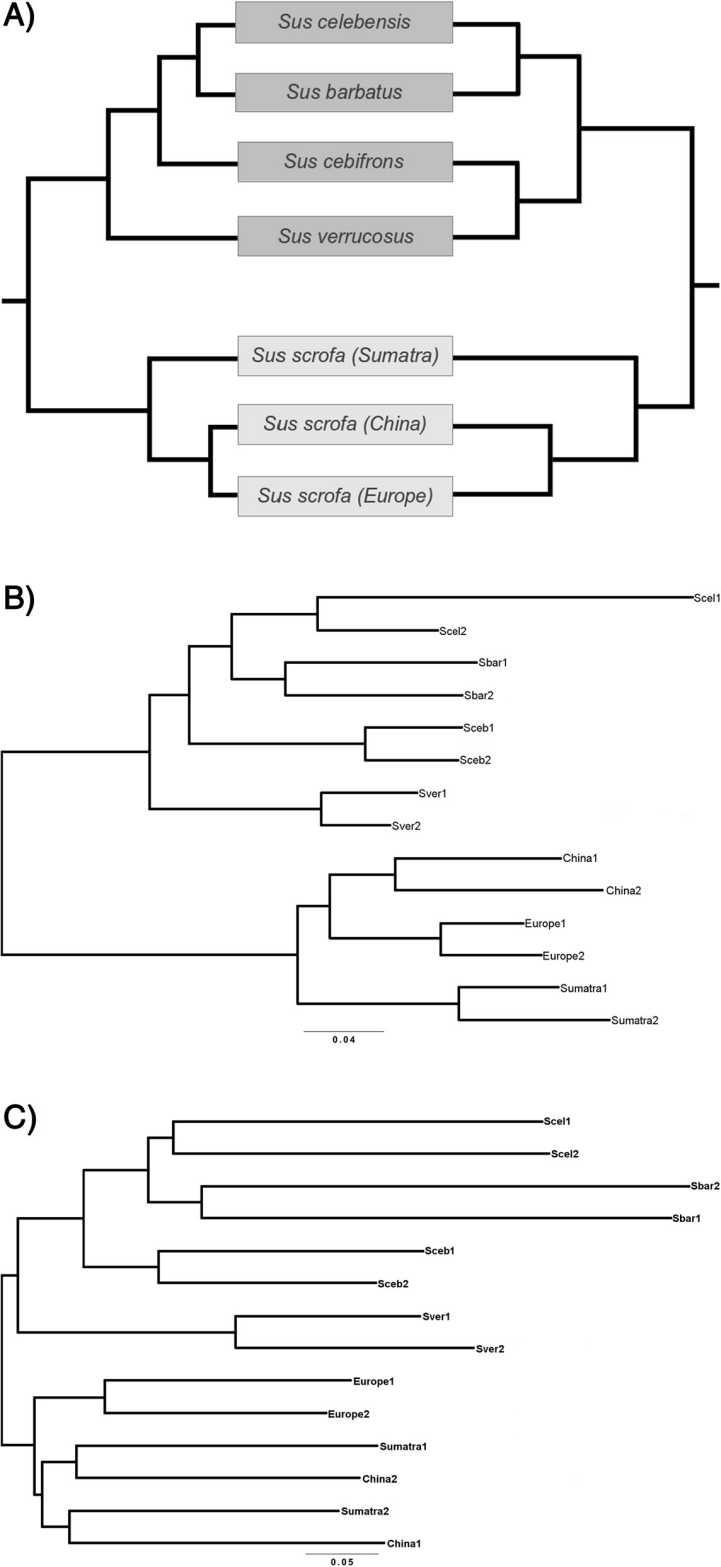
Cluster and phylogenetic tree analysis. **A**: Cluster analysis. The phylogenetic tree on the left side is obtained from Frantz et al. [31] and the cluster tree on the right side is obtained by cluster analysis using the actual CN of CNVRs from different populations. The branch length does not correspond to the evolutionary distance. **B**: NJ-Phylogenetic tree obtained by using the pairwise difference based on SNPs (Abb. see Table 1). **C**: NJ-Phylogenetic tree obtained by using the pairwise CNDs of all possible pairs for CNVRs overlapping ORs (Abb. see Table 1).

To investigate whether the observed CNVRs were congruent with the known phylogeny of the species, we performed a cluster analysis based on the CN at each CNV locus. The resulting tree is highly congruent to the phylogenomic analyses based on SNPs [31] (Figure 4A). However, some inconsistencies are observed in the resolution of branching order within *Sus*-ISEA, which is not surprising as recurring hybridization was common in the evolutionary history of *Sus*-ISEA [31].

### Rate of accumulation of CNVRs (relative to rate of accumulation of SNP)

It is generally thought that species incompatibility (e.g. through Islands of divergence) and/or lack of (intra-) species recognition are more likely to be established by fast evolving genomic regions. Thus a comparison between the rate of accumulation of CNV to other types of genetic variation, such as SNPs, could provide insight into the role of CNVs in population differentiation and speciation. To this end, a comparison between the rate of accumulation of SNPs and CNVs in each lineage was performed. To do so we first identified 1,115,908 SNPs in the genomic regions that were found to be diploid (2 copies) in all 14 individuals of 7 populations. We computed a rate of SNP accumulation, between each pair of individuals by dividing the number of observed difference with the total sites that could be confidentially called. Pairwise CNDs were obtained for all possible pairs of the 14 individuals. The CNDs were transformed into binary values with CND ≥ 2 as 1 and CND < 2 as 0. For each pair, the rate of pairwise difference was then calculated by dividing the total differences with the total CNVRs count (1408). The estimated CND rate is expected to be very conservative in comparison with the estimated rate of SNPs, due to our binary scale, which does not take into account the possible multiple changes in CN. For example, going from two to ten copies requires at least three duplication events but is considered as a single step in the current analysis. We observed that the rate of pairwise CND is approximately 2.5 times higher than the SNP rate (Additional file 4: Table S3A and S3B, respectively). The observed higher CND rate compared to the SNP rate could be the result of over-representation of ORs in the list of genes overlapping with CNVRs. To investigate this, the rate of pairwise CNDs of CNVRs overlapping with ORs and without ORs were calculated separately (Additional file 4: Table S3C and S3D). In both comparisons, i.e. CNVRs overlapping with and without ORs, the rate of pairwise CNDs was observed to be higher than for SNPs. The elevated CND rate therefore does not seem to be caused solely by expansion of the OR gene family.

### Phylogenetic analysis

The observed elevated evolutionary rate of CND may suggest that some of the CNVRs could be involved in speciation since fast evolving regions potentially play a role in the transition from pre- to postzygotic isolation. We therefore constructed neighbor joining (NJ) phylogenetic trees from SNPs and CNVRs pairwise distance matrices using PHYLIP [41]. We repeated the analysis using CNVRs overlapping with OR (CNVR-OR), CNVRs overlapping with genes other than ORs (CNVR-nonOR) and all CNVRs (CNVR-ALL). Trees obtained from SNPs (Figure 4B) and CNVR-OR (Figure 4C) resulted in nearly identical topologies. The SNP-tree topology is identical to previous phylogenomicanalysis (Figure 4A) [31] whereas the CNVR-OR-tree topology deviates slightly form the SNP-tree in the mixed relationship of the Asian *Sus Scrofa*. By contrast, phylogenetic trees obtained from CND of CNVR-nonOR (Additional file 5: Figure S2A) and CNVR-ALL (Additional file 5: Figure S2B) resulted in different topologies compared to SNP-based phylogenies where especially the CNVR-nonOR-tree topology is highly deviating from the SNP-tree. To test if population taxon sampling plays a role in the phylogenetic results, we repeated the analysis with all pairwise combinations of the four *Sus barbatus* individuals and obtained identical phylogenetic tree topologies for all different partitions (data not shown).

**Figure 5:**
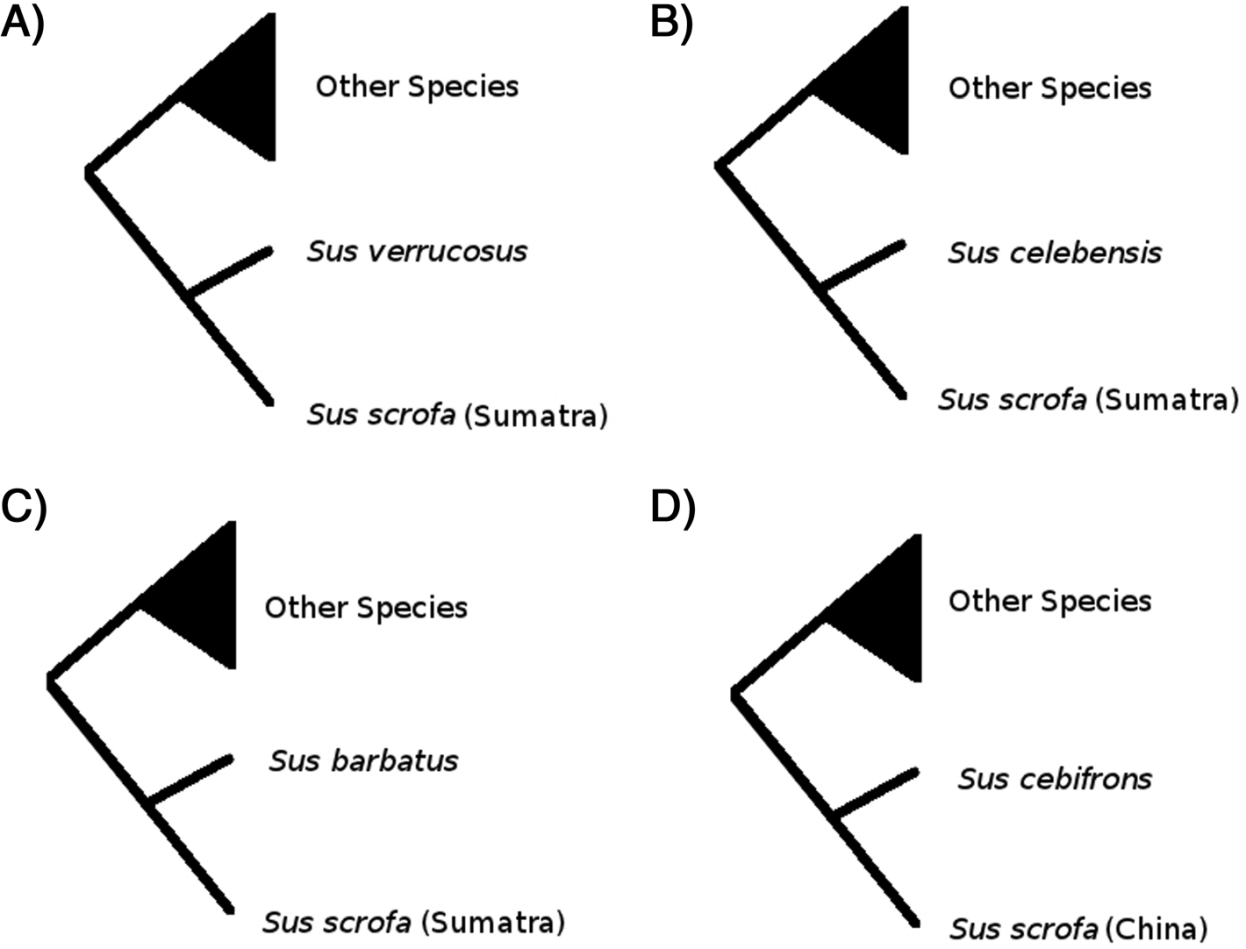
Simple schematic diagram of tested constrained models. **A**: Constrained model 1 where other species consists of *Sus scrofa* (Europe and China) and *Sus barbatus*, *Sus cebifrons* and *Sus celebensis*. **B**: Constrained model 2 where other species consists of *Sus scrofa* (Europe and China) and *Sus barbatus*, *Sus cebifrons* and *Sus verrucosus*. **C**: Constrained model 3 where other species consists of *Sus scrofa* (Europe and China) and *Sus cebifrons*, *Sus celebensis* and *Sus verrucosus*. **D**: Constrained model 4 where other species consists of *Sus scrofa* (Sumatra and Europe) and *Sus barbatus*, *Sus celebensis* and *Sus verrucosus*.

To further evaluate the discrepancies between the different partitions we performed a more parametric phylogenetic approach, Bayesian phylogenetic analysis, using the MKV model [42] as implemented in MrBayes v2.2 [43], and an extending encoding of the CNs. We first ran the MKV model without any topology constrains and found that the monophyly of the *Sus*-ISEA and *Sus scrofa* clades, as identified by the SNP data and in previous analyses [31], was highly supported (posterior probability PP > 0.9) for both CNVR-OR and CNVR-ALL, but not for CNVR-nonOR which supported a *Sus cebifons* and *Sus scrofa* (China) relationship. To address the strength of support for these discrepancies we tested different constrained models that fit the history of inter-specific admixture [31]. We first computed the support (marginal likelihood; see methods) for a null model in which the monophyly of *Sus*-ISEA and *Sus scrofa* clades were constrained, a scenario consistent with the SNP tree. Thereafter 4 different models were tested that are described in Figure 5 A-D. In Model-1, we constrained *Sus verrucosus* and *Sus scrofa* Sumatra to be monophyletic (Figure 5A), representing known admixture among these species [31]. In Model-2, we constrained *Sus celebensis* and *Sus scrofa* Sumatra to be monophyletic (Figure 5B) representing possible human translocations of *Sus celebensis* to Sumatra and neighboring islands. In Model-3, *Sus barbatus* and *Sus scrofa* Sumatra were constrained to be monophyletic (Figure 5C), representing known admixture between these two species/populations. In Model-4, *Sus cebifrons* and *Sus scrofa* China were constrained to be monophyletic (Figure 5D), representing possible migration from MSEA to the Philippines [31]. The marginal likelihood analysis strongly supports the monophyly of the two major clade of *Sus*-ISEA and *Sus scrofa* for CNVR-OR and CNVR-ALL but not for CNVR-nonOR where this monophyly provides a much poorer fit. For CNVR-nonOR the difference in marginal likelihood (delta-lnL) to the null model was 7.46 (Table 2), which strongly supports the non-monophyly of the two major clades.

**Table 2:** **Marginal likelihood scores for each partition of CNVR for different models tested**

	CNVR-ALL*	CNVR-OR*	CNVR-nonOR*
Non-constrained	7.74	7.61	6.13
Constrained (monophyly *Sus scrofa* and *Sus*-ISEA, respectively)	0	0	7.46
Constrained (*Sus scrofa* (Sumatra) and *Sus barbatus*)	47.72	16.12	21.6
Constrained (*Sus scrofa* (Sumatra) and *Sus celebensis*)	45.11	20.65	11.89
Constrained (*Sus scrofa* (Sumatra) and *Sus verrucosus*)	31.18	15.52	14.72
Constrained (*Sus scrofa* (China) and *Sus cebifrons*)	32.71	19.72	0

*delta-lnL i.e. (best marginal likelihood score) – (marginal likelihood score of the model).

### *Sus scrofa* and *Sus*-ISEA specific CNVRs

In order to identify CNVRs specific to the two monophyletic clusters, *Sus*-ISEA and *Sus scrofa* [31], we ascertained CNVRs (s.d. ≥ 0.7) in each of these clusters separately. We found 782 and 1089 CNVRs in *Sus scrofa* and *Sus*-ISEA, respectively (Additional file 6: Table S4A and S4D). A total of 687 CNVRs were found to overlap between the two groups (ascertained as CNVRs in both group) together with 98 and 407 CNVRs uniquely ascertained in *Sus scrofa* and *Sus*-ISEA group, respectively (Additional file 6: Table S4B and S4E). We observed 243 genes in the 687 CNVRs whereas uniquely ascertained CNVRs in *Sus scrofa* and *Sus*-ISEA contained 47 and 178 genes, respectively (Additional file 6: Table S4C and S4F). Most of the genes unique to each cluster were found to be OR genes. Notable, the majority of the OR genes that were observed to vary in *Sus*-ISEA were found to be fixed with high CN in *Sus scrofa* populations. To test if taxon sampling introduces a bias in these group specific analyses (because of four populations in *Sus*-ISEA and three in *Sus scrofa*), we re-sampled every possible combination of three in the *Sus*-ISEA cluster. This sampling correction did not affect any of the results described above (e.g. there was always a higher number of CNVRs in *Sus*-ISEA than *Sus scrofa*; number of CNVRs in *Sus*-ISEA group varied from 917 to 1026).

## Discussion

### Evolution of CNVRs in the genus *Sus* and their possible role in the on-going *Sus* speciation process

The comparison between the seven populations of genus *Sus* (two of them (*Sus cebifrons* and *Sus verrucosus*) are listed as threatened species [34]) allowed us to elucidate general and species-specific features of CNVs. It is known that compared to SNPs, CNVRs cover a larger part of the genome (in terms of nucleotides) and potentially have larger effects by, for example, changing gene structure, gene dosage and alternating gene regulation [44,45]. In this study, we detected 1408 CNVRs in these five closely related species of the genus *Sus*. The functional enrichment analysis of the CNVRs suggested that genes involved in sensory perception of smell, signal transduction, neurological process, and metabolic process are over-represented in CNVRs. The most abundant gene family in the porcine genome, the OR gene family, was observed as highly over-represented in the CNVRs. This over-representation of OR genes in the CNVRs could have strong functional consequences since pigs strongly rely on their sense of smell for finding food, predators, and most importantly potential mates.

The process of (on-going) speciation is thought to be triggered by a combination of many different mechanisms which include processes such as, gradual adaptation to different environment, evolution of divergent mate recognition and other molecular mechanism which are thought to be influenced by fast evolving regions in the genome. These fast evolving regions potentially accumulate divergence faster, which eventually result in creating reproductive barriers between populations. CNVRs can be a major mechanism driving gene and genome evolution by duplication and deletion of segments of the genome and as a result, create novel gene functions, disrupt gene functions, or affect regulatory mechanisms in the genome. The comparison between the rate of accumulation of CNVRs and the rate of accumulation of SNPs suggests that the CNVRs are evolving approximately 2.5 fold faster than SNPs, which is in line with a recent study in apes [46] where a 1.4 fold differences was observed between CNVRs and SNPs. Thus, these fast evolving CNVRs, especially those overlapping with functional regions in the genome might be a major driver of the on-going speciation in pigs.

The recent study on speciation of the genus *Sus* has shown that these taxa have undergone multiple rounds of small-scale inter-specific hybridization (i.e. admixture) during the glacial periods of the Pleistocene (2.5-0.01 Mya) [31]. Despite the multiple events of interspecific hybridization and being geographically very close to *Sus*-ISEA populations, the Sumatran *Sus scrofa* population (found to be coexisting with *Sus barbatus* on Sumatra) was found to be less admixed with *Sus*-ISEA than *Sus scrofa*. This implies the existence of mechanisms that prevented these species from massive homogenizing during the numerous glacial periods of the Pleistocene. Furthermore, the phylogenetic tree analysis based on pairwise CND of CNVR-OR and pairwise difference in SNPs suggests that CNVR-OR largely recapitulates the accepted phylogeny of the genus *Sus* [31], whereas the phylogenetic trees obtained by using pairwise CND of CNVR-nonOR, show inconsistencies with the phylogenetic history of the genus *Sus* and instead follows expected patterns of random drift and/or admixture [31] (Additional file 5: Figure S2A and B). The strength of support for these inconsistencies were assessed by testing the support of different constrained models that fit the history of inter-specific admixture reported in a previous study [31] using a novel Bayesian phylogenetic analysis approach. The Bayesian phylogenetic analysis on the CN partitions significantly supported the recapitulations of topology of the genus *Sus* by CNVR-OR whereas for CNVR-nonOR the inconsistent topology representing admixture/random drift of genus *Sus* was strongly supported. Thus, CNVRs with OR show resistance to admixture and random drift effects between the analyzed species. This observation in combination with the observed higher rate of evolution suggests that these OR genes could play a major role in the on-going speciation process of *Sus*, facilitating rapid adaptation to different environments and divergence in mate recognition. Furthermore, pigs are known to depend highly on their sense of smell for foraging and mate recognition, and have one of the largest functional OR repertoires observed in mammals, which additionally makes it plausible that ORs are important in speciation of pigs.

Besides OR genes, genes involved in immune response, defense to pathogens and detoxification such as interferons (*IFN*), *NPG3*, *PMAP23* and cytochrome P450 (*CYP*), are usually also fast evolving due to their importance for the organism to respond rapidly to changes in the environment and food-borne pathogens [26,35,36,38,46,47]. Thus, together with ORs, the observed variation in CN of these genes suggests an ongoing process of evolution of these gene families and their importance for adaptation in a rapidly changing environment.

Despite the similar divergence time [31], the total CNVRs in the *Sus*-ISEA group (1089; 407 specific to *Sus*-ISEA) was found to be higher than that in *Sus scrofa* (782; 96 specific to *Sus scrofa*). In addition, for the 407 *Sus*-ISEA specific CNVRs, *Sus scrofa* shows universal high and fixed CN between three diverse *Sus scrofa* populations and most of the genes overlapping with group specific CNVRs are found to be ORs (178 genes; 146 ORs). This fixation might have happened soon after the split of the ancestral *Sus scrofa* population from the other *Sus* species from ISEA around 4 Mya.

We suggest that CNVR-ORs, might have provided the means to rapid adaption to different environments during the diversification of the genus in the Pliocene [31]. Further, the CNVR-ORs might have acted as barriers against gene flow during the multiple round of hybridization that took place later in the Pleistocene. To what extent these regions might have played a role in differentiating of *Sus scrofa* from the rest of the suids is another interesting topic which requires a more extensive taxon sampling of highly diverged suids from other parts of the world.

## Conclusions

We identified 1408 CNVRs across the genus *Sus*. These CNVRs encompass 624 genes and were found to evolve ~2.5 times faster than SNPs. The majority of these copy number variable genes are ORs known to play a prominent role in food foraging and mate recognition in *Sus*. Phylogenetic analyses, including novel Bayesian analysis, based on CNVRs that overlap ORs retain the well-accepted topology of the genus *Sus* whereas CNVRs overlapping genes other than ORs show evidence for random drift and/or admixture. We hypothesize that inter-specific variation in copy number of ORs provided the means for rapid adaptation to different environments during the diversification of the genus *Sus* in the Pliocene. Furthermore, these regions might have acted as barriers preventing massive gene flow between these species during the multiple hybridization events that took place later in the Pleistocene suggesting a possible prominent role of ORs in the ongoing *Sus* speciation.

## Methods

### Samples and data generation

In total 16 different individuals from 5 different species were sequenced using the Illumina platform (Illumina GAII or HiSeq, Illumina, San Diego, CA, USA). The sequences are 100 bases pair-end reads from 400–500 bp insert-libraries with coverage per animal ranging between 7 – 18x. The sampled pigs comprised of European wild boar (2- Dutch, *Sus scrofa*), Chinese wild boar (2- South Chinese, *Sus scrofa*), Sumatran wild boar (2- Sumatra, *Sus scrofa*), *Sus barbatus* (4 individuals), *Sus cebifrons* (2 individuals), *Sus celebensis* (2-individuals) and *Sus verrucosus* (2 individuals) (Table 1; Additional file 1: Table S1A). Blood samples were obtained from veterinarians according to national legislation and tissue samples were obtained from animals culled within wildlife management programs. DNA from blood or tissue was extracted using the DNeasy blood and tissue kits (Qiagen, Venlo, NL, USA). Quality and quantity were measured with the Qubit 2.0 Fluorometer (Life Technologies, Carlsbad, CA, USA).

### Sequence alignment and copy number estimation

The CN of regions in the genomes of all individuals was detected by a RD method [35,38,48], where the number of copies is inferred from sequence depth of whole genome sequence data. To calculate the average read depth from those libraries, reads were first aligned to the repeat masked reference genome (*Sus scrofa* build 10.2) using mrsFAST v2.3.0.2 (“Micro-read (substitutions only) fast alignment and search tool” [49]) with an edit distance of at most 7 given that the mean divergence between the seven species is maximum 2% [39,31]. Repeat masked information was obtained from NCBI (reference genome and repeat masked reference genome: ftp://ftp.ncbi.nih.gov/genbank/genomes/Eukaryotes/vertebrates_mammals/Sus_scrofa/Sscrofa10.2/Primary_Assembly/assembled_chromosomes/FASTA/) and merged with the repeat masked information used in Groenen et al. [39]. Because the RD methods do not take paired-end information into consideration, all the paired-end sequences were treated as single-end sequences. Two individuals from each species were merged and treated as one to increase the confidence and sensitivity to infer CN (see results). Calculation of read depth across the whole genome was done with the help of SAMtools v0.1.18 (r982:295) [50]. Average read depth for each 1 Kb non-overlapping bins of repeat masked genome was calculated. To be considered for further analysis, a bin needs to have at least 300 bases of unmasked region.

The RD method uses read depth information of diploid regions as the reference to infer CN. Since no prior information regarding diploid regions in the porcine genome was available, we initially used 1:1 orthologous genic regions between human, cow and pig and assumed these to be diploid in pig to identify CN of each 1Kb bin present in the genome. Because coding regions are known to have a higher GC content than the genome average [51,52] this procedure may introduce a GC biased read depth. Hence, to reduce possible GC bias introduced by the 1:1 orthologous regions, all diploid regions predicted from 1:1 orthologous regions in the first stage were subsequently used to recalculate the average diploid read depth of the porcine genome as described previously [38].

Next generation sequencing methods have been shown biased in coverage in regions of high or low GC [53-58]. To correct for this bias we calculated GC intervals correction factors as described by Sudmant et al. [35]. These factors were then used to correct read depth of each 1 Kb bin across the genome. CN of each 1 Kb non-overlapping bin was then estimated based on the GC corrected read depth. Since the samples include both male and female individuals, sex chromosomes were excluded from the analysis.

### Prediction of MCRs and defining CNVRs

All the 1 Kb bins with minimum CN of 1 were extracted from all individuals and bins with CN ≥2.5 were chained to form multi copy regions (MCRs). The same MCRs might be assigned with different boundaries in different individuals due to technical and/or biological reasons. Therefore, all the MCRs from all individuals were extracted, merged, and CN of those regions for all individuals were calculated and compared. Further, the MCRs with standard deviation of CN higher than 0.7 (s.d. ≥0.7) between all individuals were assigned as CNVRs [38].

### Gene identification and Gene Ontology

All the annotated porcine genes from *Sus scrofa* build 10.2, Ensembl release 75, were extracted using BioMart [59] and genes overlapping with the CNVRs (≥70% overlap) were identified. Not all pig genes have associated gene names, thus the genes without gene names were aligned against the human Refseq mRNAs and human reference protein sequences (blastn and blastp, respectively), and the best human hit was assigned as gene name. Human orthologs of porcine genes were then used to perform a gene ontology analysis. BinGO v2.44 [60] a plugin of Cytoscape v2.8.3 [61] was used to identify enriched GO terms using human gene annotation as background. A hypergeometric test was used to assess the significance of the enriched terms and Benjamini-Hochberg FDR correction was implemented for multiple comparisons.

### *Sus scrofa* specific and other suids specific CNVRs

For the group comparison, we formed two groups: one with *Sus scrofa* including all three diverse populations of *Sus scrofa* and another with the *Sus*-ISEA. CNVRs for both groups were generated based on the similar approach described above comparing only individuals belonging to a group.

### Cluster analysis

Hierarchical cluster analysis was performed using R package “hclust” on the CN at each CNVR. Initially, each species is assigned to its own cluster and then the algorithm proceeds iteratively, at each CNVR joining the two most similar clusters, continuing until there is just a single cluster.

### SNP calling

SNPs were called in each individual of a population separately. We extracted all the regions that were assigned as diploid (CN 2) in all populations. We then used Samtools v0.1.19 mpileup [50] to call genotype at sites and only considered genotype calls as SNPs, if they are different from the reference base and covered by at least 4 reads with minimum base and mapping quality of 20.

### Estimation of pairwise distance between SNPs and CNVRs and construction of phylogenetic tree

A rate of SNP accumulation, between all possible pair of the 14 individuals was computed by dividing the number of observed difference with the total sites that could be called confidently i.e. 1,115,908 SNPs. The CNDs were transformed into binary values with CND ≥ 2 as 1 and CND < 2 as 0. For each pair, the rate of pairwise difference was then calculated by dividing the total differences with the total CNVRs count (1408). PHYLIP package v3.695 [41] was used to construct neighbor joining (NJ) phylogenetic trees from the calculated pairwise distance matrix of SNPs and the following partitions of CNVRs: CNVR-OR (CNVRs overlapping OR genes) CNVR-nonOR (CNVRs overlapping non-OR genes) and CNVR-ALL (all CNVRs with and without gene overlap).

### Construction of phylogenetic trees using a Bayesian approach

Bayesian phylogenetic analysis was performed using the MKV model [42] as implemented in MrBayes v2.2 [43]. This model implements a maximum likelihood approach to variable characters (i.e. morphology). To use this model with our CN data we need discrete CN values between 0 and 9. We used the following equation to transform CNs of each locus for each species into 9 discrete values.

$$ {\mathrm{CN}}_{\mathrm{n}} = \left(\left({\mathrm{CN}}_{\mathrm{o}} - {\mathrm{CN}}_{\min}\right)/\left({\mathrm{CN}}_{\max}\hbox{--}\ {\mathrm{CN}}_{\min}\right)\right)*\left(10\hbox{--} 1\right) $$

where, CN_n_ = Transformed CN_n_ (rounded)

CN_o_ = Raw CN

CN_max_ = Maximum observed CN for locus

CN_min_ = Minimum observed CN for a locus

We used the default (infinity) hyper-prior for the dirchelet process that model rate classes. This model implies little variation among rate of transition between CN. More complex models can be used by decreasing the hyper-prior (increasing concentration parameter). However, because increasing the concentration parameter (the number of rate categories) for the dirichelet process greatly increases the running speed, we kept this parameter to the default settings. For each data set (CNVR-OR, CNVR-nonOR and CNVR-ALL) we first ran 1,000,000 Markov Chain Monte Carlo (MCMC) (25% burnin) samples to estimate posterior distributions of the various parameters. Marginal likelihoods were computed using the stepping-stone model [62,63] with 1,000,000 samples (25% burnin) and 50 steps. We also estimated the marginal likelihood under different constrained models (see Results) to further investigate the support for discrepancies found among data sets and between NJ and Bayesian trees.

### qPCR Validation

Primer3 webtool http://frodo.wi.mit.edu/primer3/ was used to design primers for qPCR validation. Amplicon length was limited between 50 bp to 100 bp and regions with GC percentage between 30% and 60% were included, while avoiding runs of identical nucleotides. All other settings were left at their default. Details of the qPCR primers can be found in Additional file 6: Table S4G. qPCR experiments were conducted using MESA Blue qPCR MasterMix Plus for SYBR Assay Low ROX from Eurogentec, this 2x reaction buffer was used in a total reaction volume of 12.5 μl. All reactions were amplified on 7500 Real Time PCR system (Applied Biosystems group). The CNDs were determined by using a standard ∆Ct method that compares the mean Ct value of the target CND fragments, determined from different input concentrations, compared to the mean Ct value of a known diploid reference.

### Availability of supporting data section

European Nucleotide Archive: ERP001813.

## Additional files

Additional file 1: Table S1. A: List of species and animals with their sequence depth including information of additional 2 *Sus barbatus* samples. B: List of CNVRs with all species. C: List of CNVRs with CN of each individual separately including additional *Sus barbatus* samples. D: General statistic of shared CNVRs between species. E: qPCR results for the twenty validated CNVRs. Additional file 2: Figure S1. Heatmap of CNVRs in all chromosomes. A) Heatmap with combined CNVRs in all chromosomes. Each column represents a population (combined CN) and each row represents a CNVR. B) Heatmap with of CNVRs in all chromosomes. Each column represents an individual separately (CN in that individual only) and each row represents a CNVR and genes overlapping with the CNVRs are listed next to the CNVRs (right). Additional file 3: Table S2. A: List of genes overlapped by CNVRs. B: List of olfactory receptor genes overlapped by CNVRs. C: Gene ontology using BinGO package. Additional file 4: Table S3. A: List of species and animals with their sequence depth including information of additional 2 *Sus barbatus* samples. B: List of CNVRs with all species. C: List of CNVRs with CN of each individual separately including additional *Sus barbatus* samples. D: General statistic of shared CNVRs between species. E: qPCR results for the twenty validated CNVRs. Additional file 5: Figure S2. Phylogenetic trees. A) Phylogenetic trees obtained from CND of CNVR-nonOR. B) Phylogenetic trees obtained from CND of all CNVR. Additional file 6: Table S4. A) CNVRs in *Sus scrofa* lineage. B) List of uniquely ascertained CNVRs in *Sus scrofa* lineage. C) List of genes in uniquely ascertained CNVRs in *Sus.* D) CNVRs in *Sus*-ISEA lineage. E) List of uniquely ascertained CNVRs in *Sus*-ISEA lineage. F) List of genes in uniquely ascertained CNVRs in *Sus*-ISEA. G) List of qPCR primers.

## Abbreviations

SV: Structural variation
MCR: Multi copy region
CNV: Copy number variation
CNVR: Copy number variable region
CND: Copy number difference
OR: Olfactory receptor
ISEA: Islands of South East Asia
MSEA: Main land South East Asia
