# Supplementary figures and images for: Copy number variation in the speciation of pigs: a possible prominent role for olfactory receptors
Source: BMC Genomics. 2015 Apr 22;16(1):330. doi: 10.1186/s12864-015-1449-9 (PMC4413995; doi:10.1186/s12864-015-1449-9)

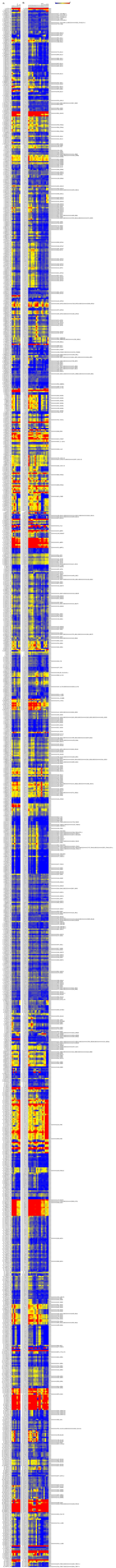

Supplement: Additional file 2: Figure S1. — Heatmap of CNVRs in all chromosomes. A) Heatmap with combined CNVRs in all chromosomes. Each column represents a population (combined CN) and each row represents a CNVR. B) Heatmap with of CNVRs in all chromosomes. Each column represents an individual separately (CN in that individual only) and each row represents a CNVR and genes overlapping with the CNVRs are listed next to the CNVRs (right). [file 12864_2015_1449_MOESM2_ESM.jpeg]

A)

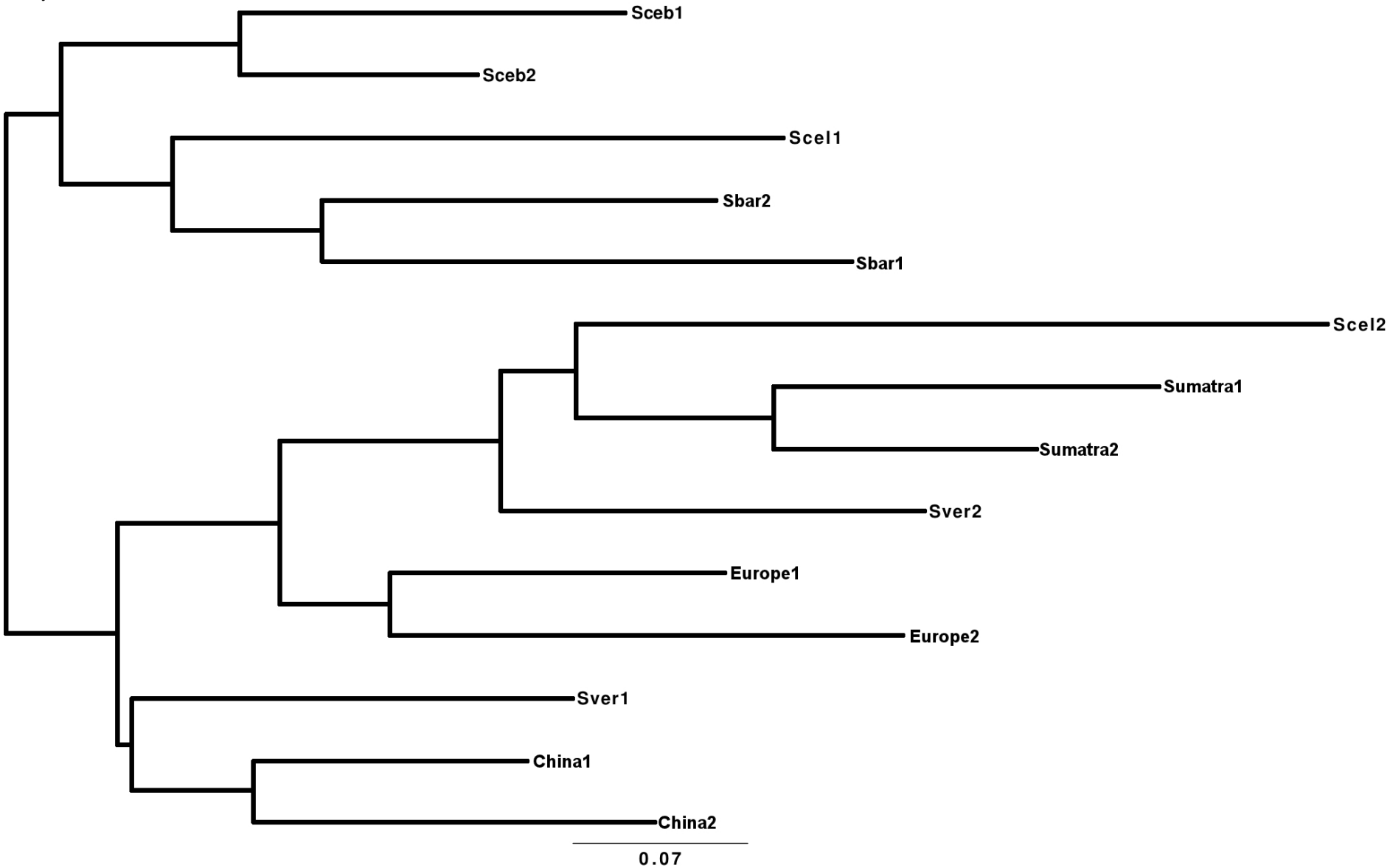

B)

SceI2

SceI1

Sbar2

Sbar1

Sceb1

Sceb2

China1

China2

Sver1

Sver2

Sumatra1

Sumatra2

Europe1

Europe2

0.05

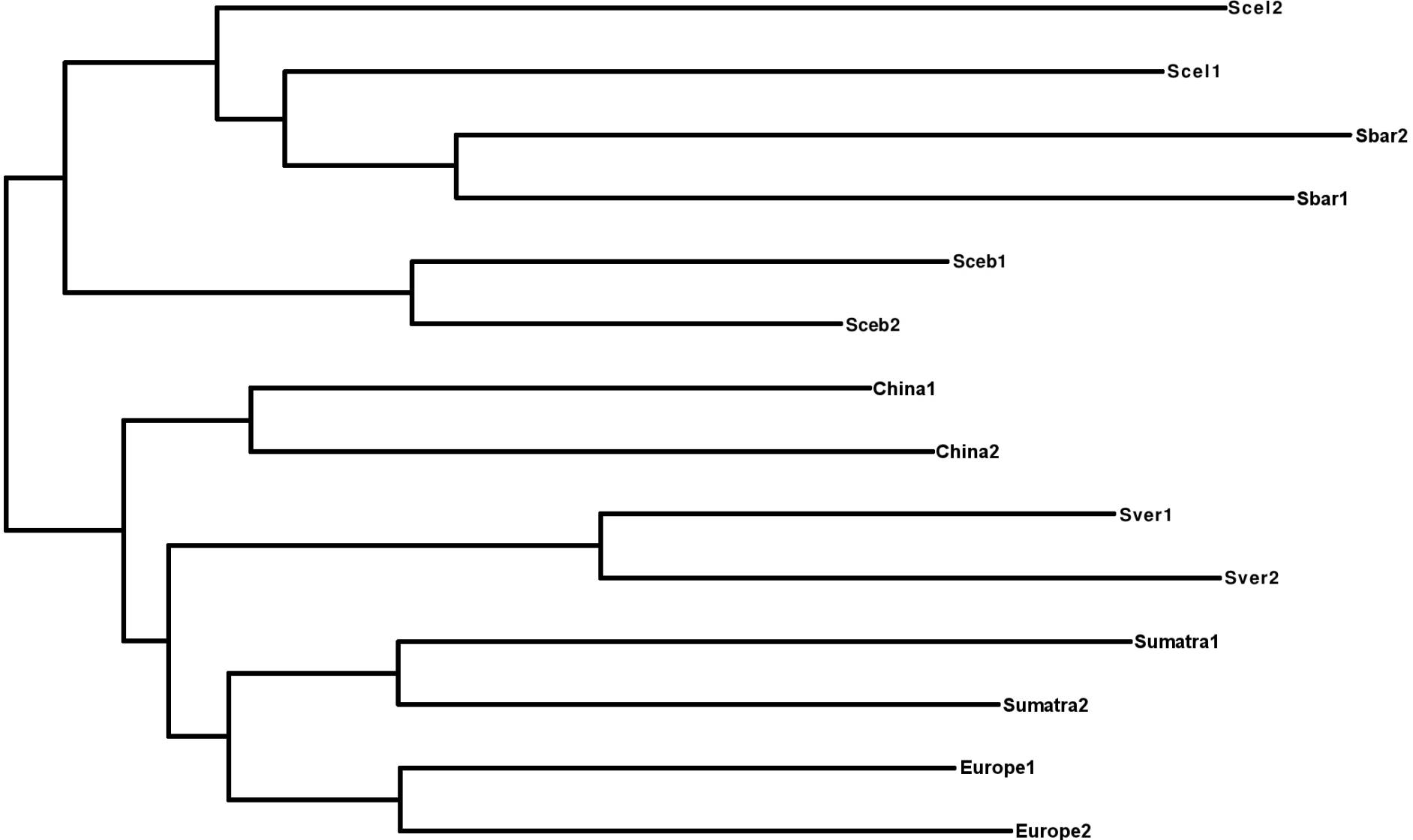

Supplement: Additional file 5: Figure S2. — Phylogenetic trees. A) Phylogenetic trees obtained from CND of CNVR-nonOR. B) Phylogenetic trees obtained from CND of all CNVR. [file 12864_2015_1449_MOESM5_ESM.pdf]
